# Supplementary material for: Antarctic Peninsula warm winters influenced by Tasman Sea temperatures
Source: Nat Commun. 2021 Mar 8;12:1497. doi: 10.1038/s41467-021-21773-5 (PMC7940433; doi:10.1038/s41467-021-21773-5)
Supplement: Supplementary file 1 — Supplementary Information [file 41467_2021_21773_MOESM1_ESM.pdf]

Supplementary Information

Antarctic Peninsula warm winters influenced by Tasman Sea temperatures

Kazutoshi Sato[1,2]*, Jun Inoue[3,4,2], Ian Simmonds[5] and Irina Rudeva [6,5]

[1]Kitami Institute of Technology, Kitami, Japan; [2]Application Laboratory, Japan Agency for Marine-Earth Science and Technology, Yokohama, Japan; [3]National Institute of Polar Research, Tachikawa, Japan; [4]The Graduate University for Advanced Studies, SOKENDAI, Hayama, Japan; [5]School of Earth Sciences, The University of Melbourne, Melbourne, Australia; [6]Australian Bureau of Meteorology, Melbourne, Australia

Including:

Supplementary Fig. 1.
Supplementary Fig. 2.
Supplementary Fig. 3.
Supplementary Fig. 4.
Supplementary Fig. 5.
Supplementary Fig. 6.
Supplementary Fig. 7.
Supplementary Fig. 8.
Supplementary Fig. 9.
Supplementary Fig. 10.
Supplementary Fig. 11.
Supplementary Fig. 12.
Supplementary Fig. 13.
Supplementary References

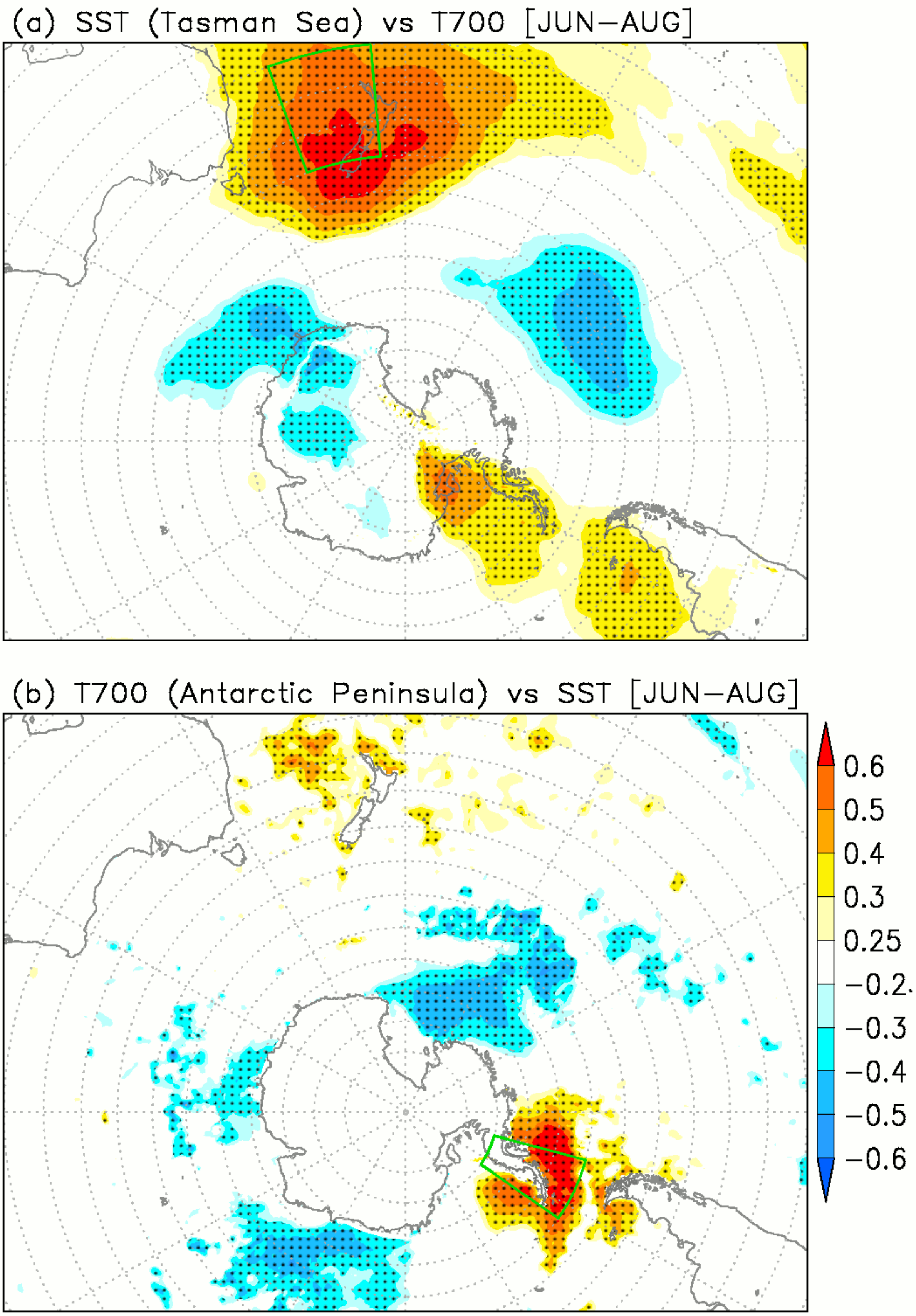

Supplementary Fig. 1. Relationships between Sea Surface Temperature and Temperature at 700 hPa over the Southern Hemisphere during June to August. Map of correlation coefficients (a) between in sea surface temperature (SST) over the the Tasman Sea (green box) and air temperature at 700 hPa (T700), and **(b)** between T700 over the Antarctic Peninsula (green box) and SST. Dotted areas denote significant differences exceeding the 95% confidence level.

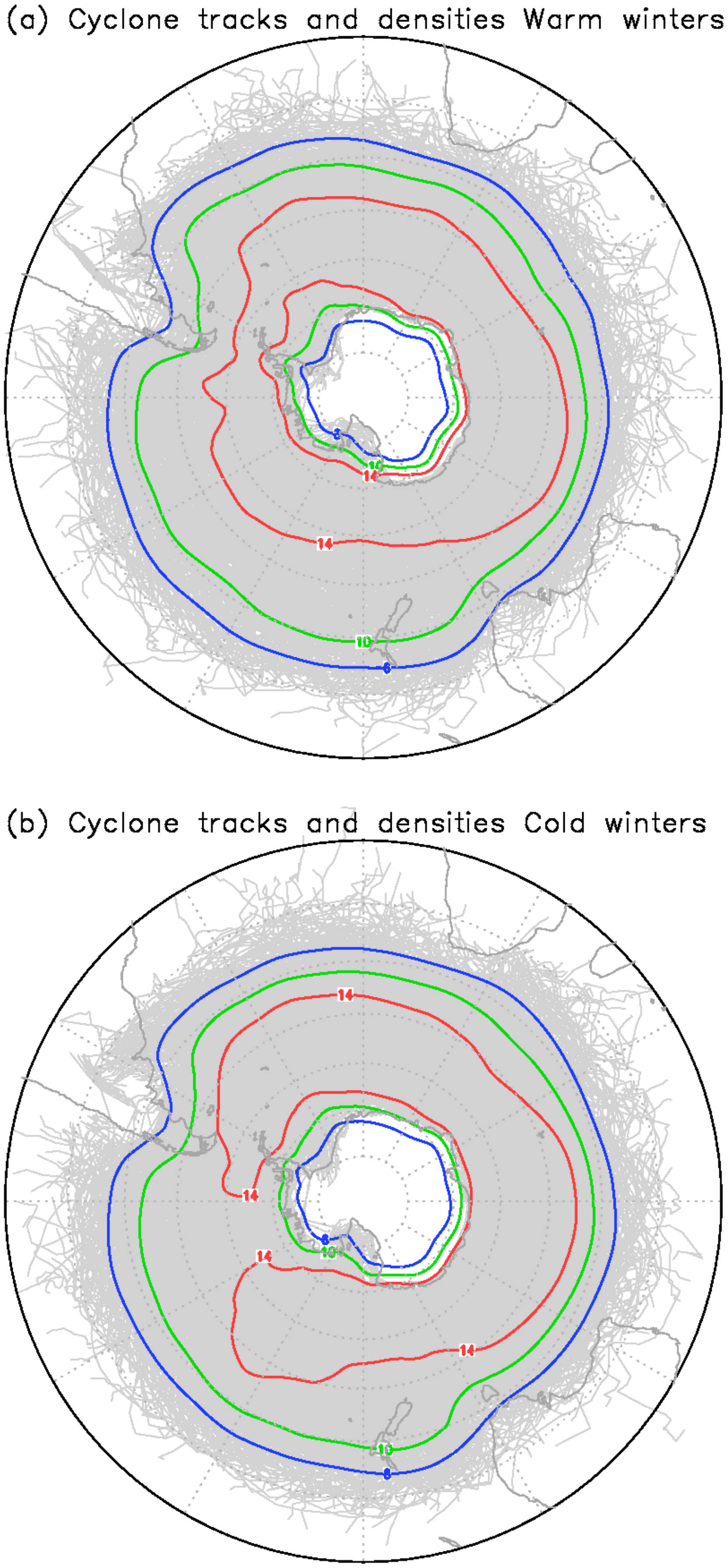

Supplementary Fig. 2. Cyclone density and tracks for warm and cold winters. Composite densities of cyclones (colour contours: count/S) and cyclone tracks (grey lines) for (a) warm and (b) cold winters.

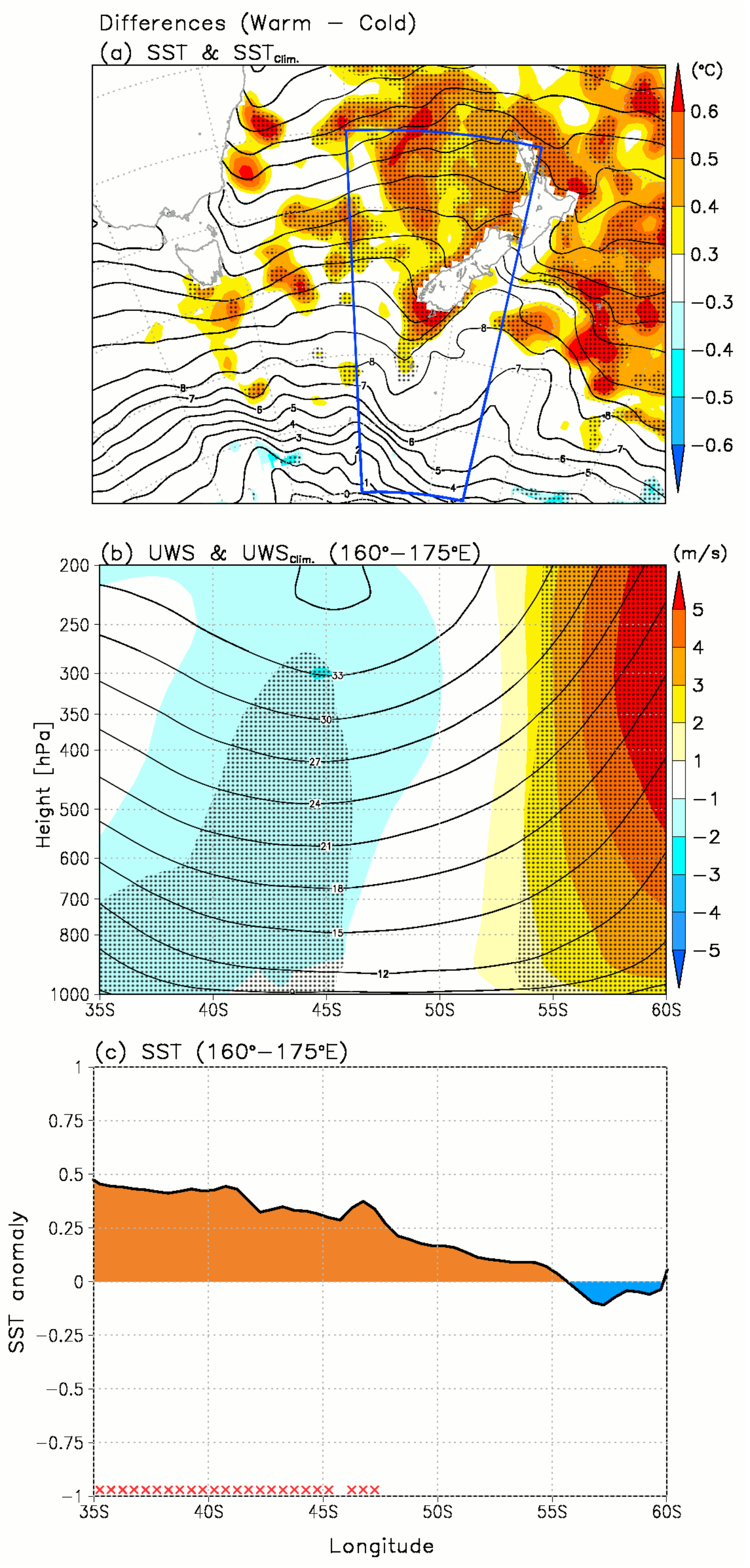

Supplementary Fig. 3. Atmospheric and oceanic differences over the Tasman Sea. (a) Difference maps of sea surface temperature (SST) between warm and cold winters (°C: shaded) with SST for warm winters (°C: contour) in the Tasman Sea region. (b) Difference of U-wind speed (UWS) (m/s: shaded) between warm and cold winters with UWS (m/s: contour) for warm winters as a function of latitude averaged over the blue rectangular area. Dotted areas indicate domains over which the differences are significant at the 95% confidence level. (c) Difference in SST as a function of latitude, averaged between 160°E and 175°E within the area of the blue rectangle (35°S-60°S, 160°E-175°E). Red crosses indicate latitudes over which the differences are significant at the 95% confidence level.

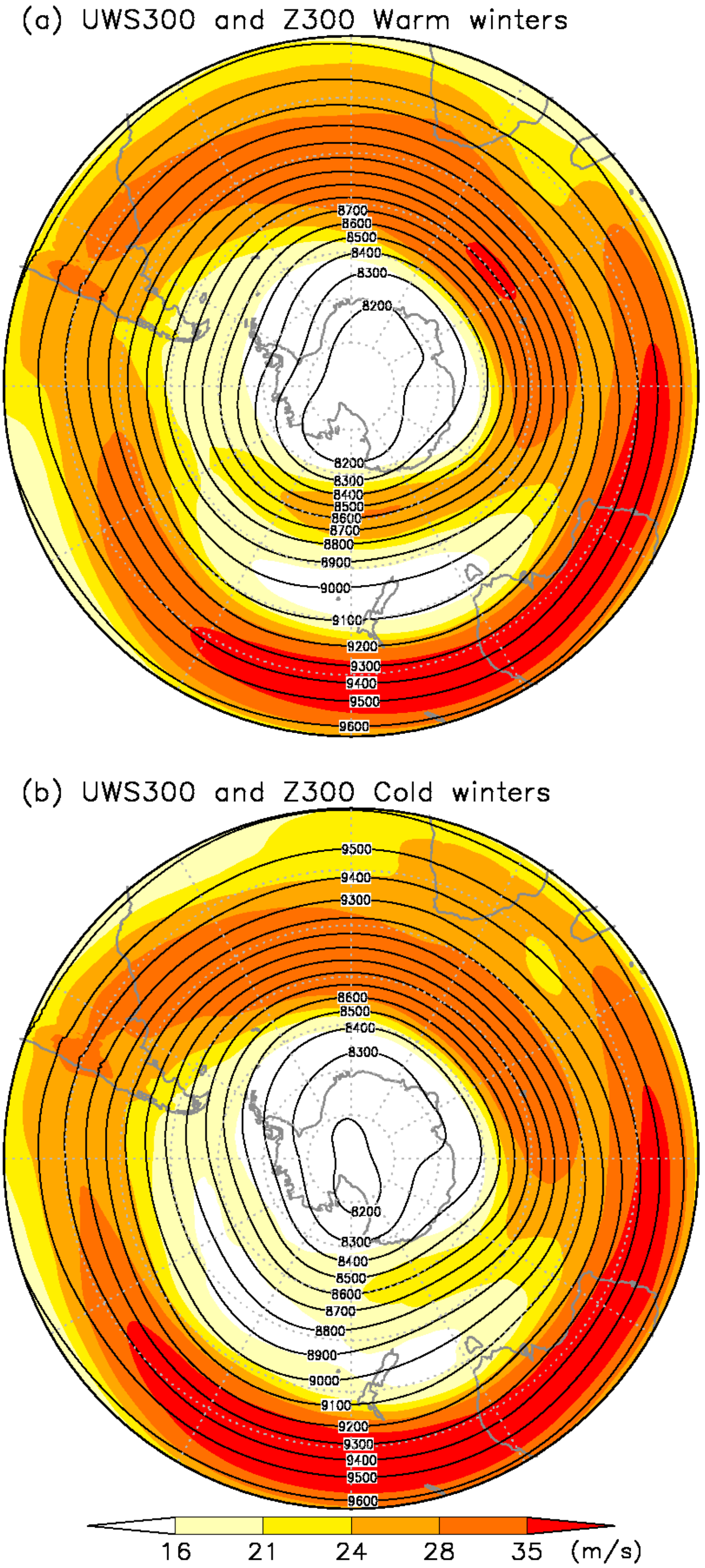

Supplementary Fig. 4. U-wind speed and geopotential height at 300 hPa. Composite U-wind speed at 300 hPa (UWS300) (m/s: shading) and geopotential height at 300 hPa (Z300) (m: colour contours) for (a) warm and (b) cold winters.

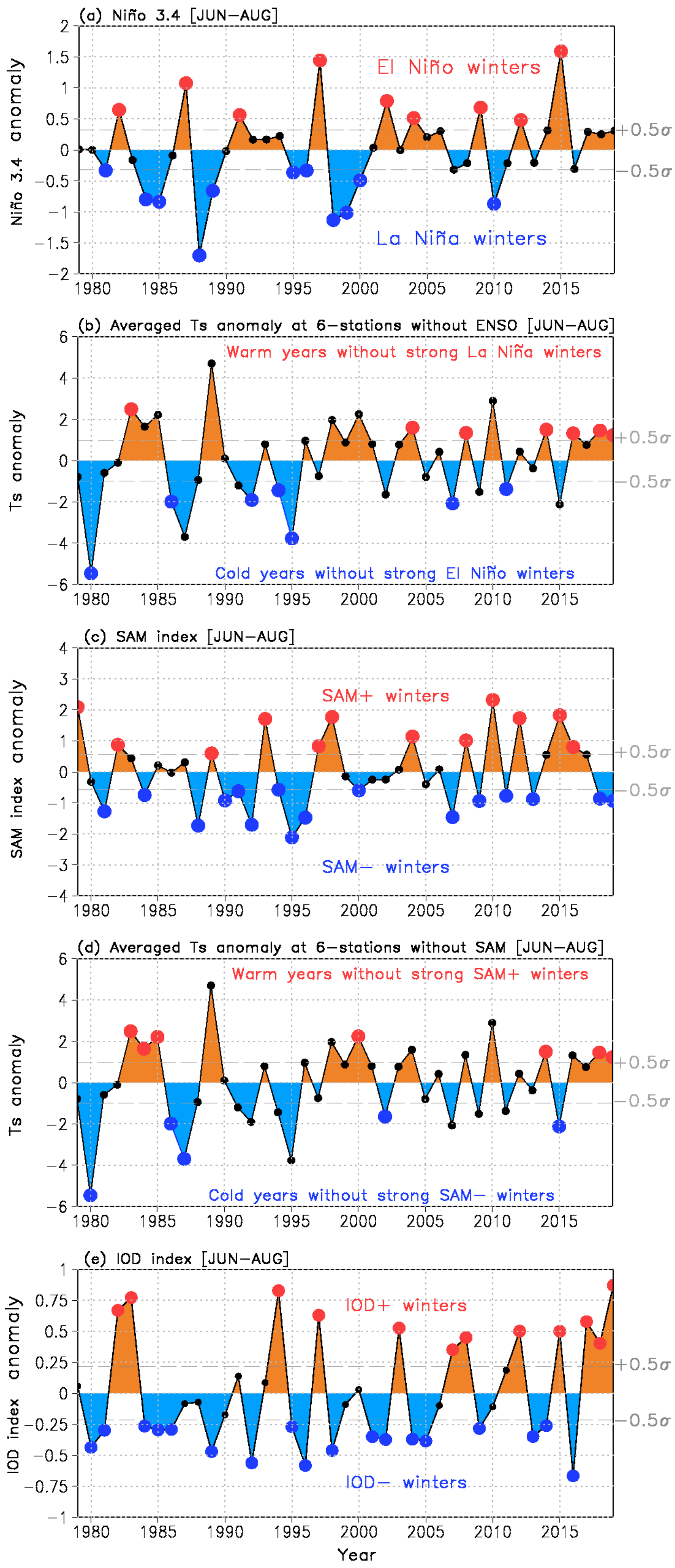

Supplementary Fig. 5. Time series of (a) the NINO3.4 index during June to August. Red and blue dots indicate strong El Niño and strong La Niña winters, respectively. (c) (e) Same as (a), but for the SAM index (c) and IOD index (e). (b) Time series of averaged surface air temperature anomalies (°C, deviation from climatology for 1979–2019) during June and August at six AP stations. Red and blue dots indicate the warm and cold winters for which the magnitudes of the temperature anomaly values exceeded one half standard deviation without strong El Niño or La Niña. (d) Same as (b), but without positive and negative winters. Dashed lines show one half standard deviation (±0.5σ).

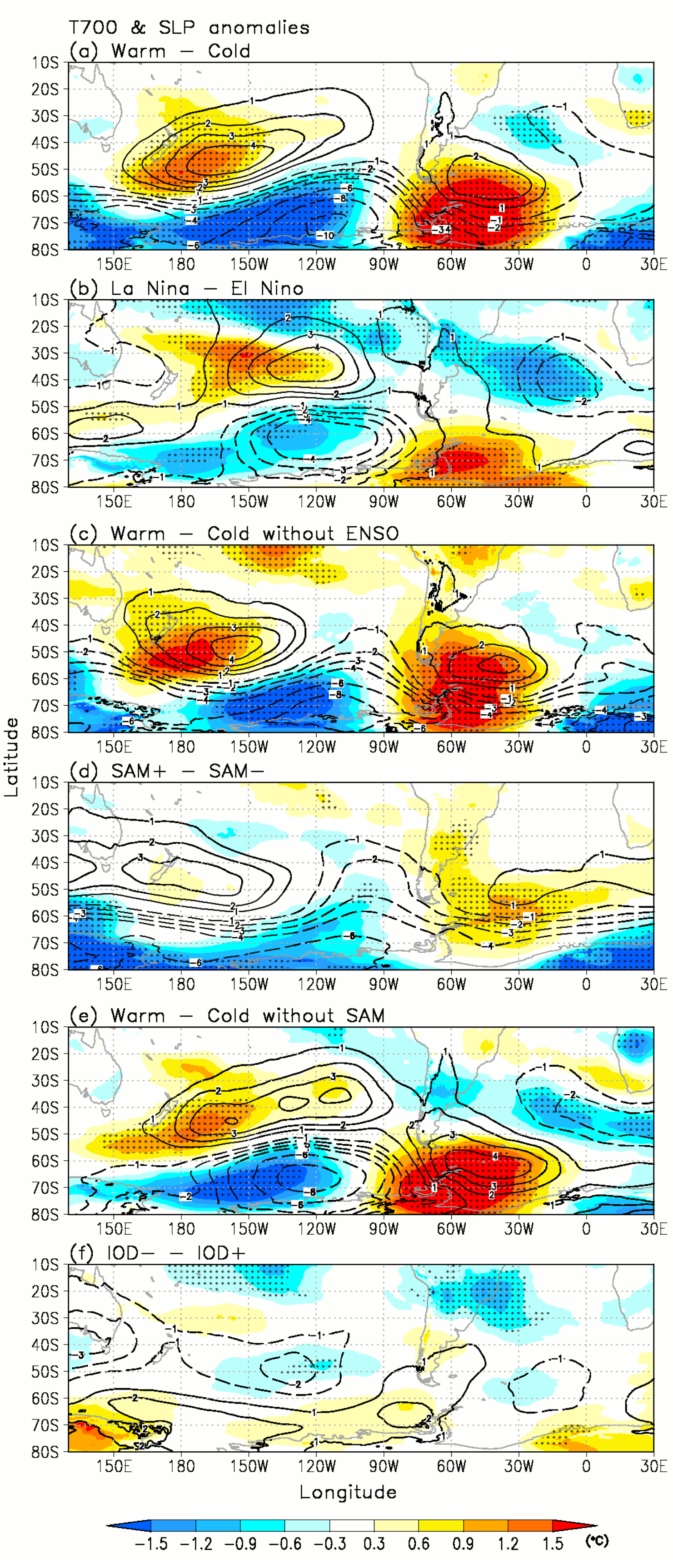

Supplementary Fig. 6. Temperature and sea level pressure anomalies. Difference maps for air temperature at 700 hPa (T700) (°C: shaded) and sea level pressure (SLP) (hPa: contours) for (a) between warm and cold winters, (b) between La Niña and El Niño winters, (c) between warm and cold winters without strong ENSO, (d) between positive SAM and negative SAM winters, (e) between warm and cold winters without strong SAM and (f) between negative IOD and positive IOD winters. Dotted areas denote significant differences exceeding the 95% confidence level.

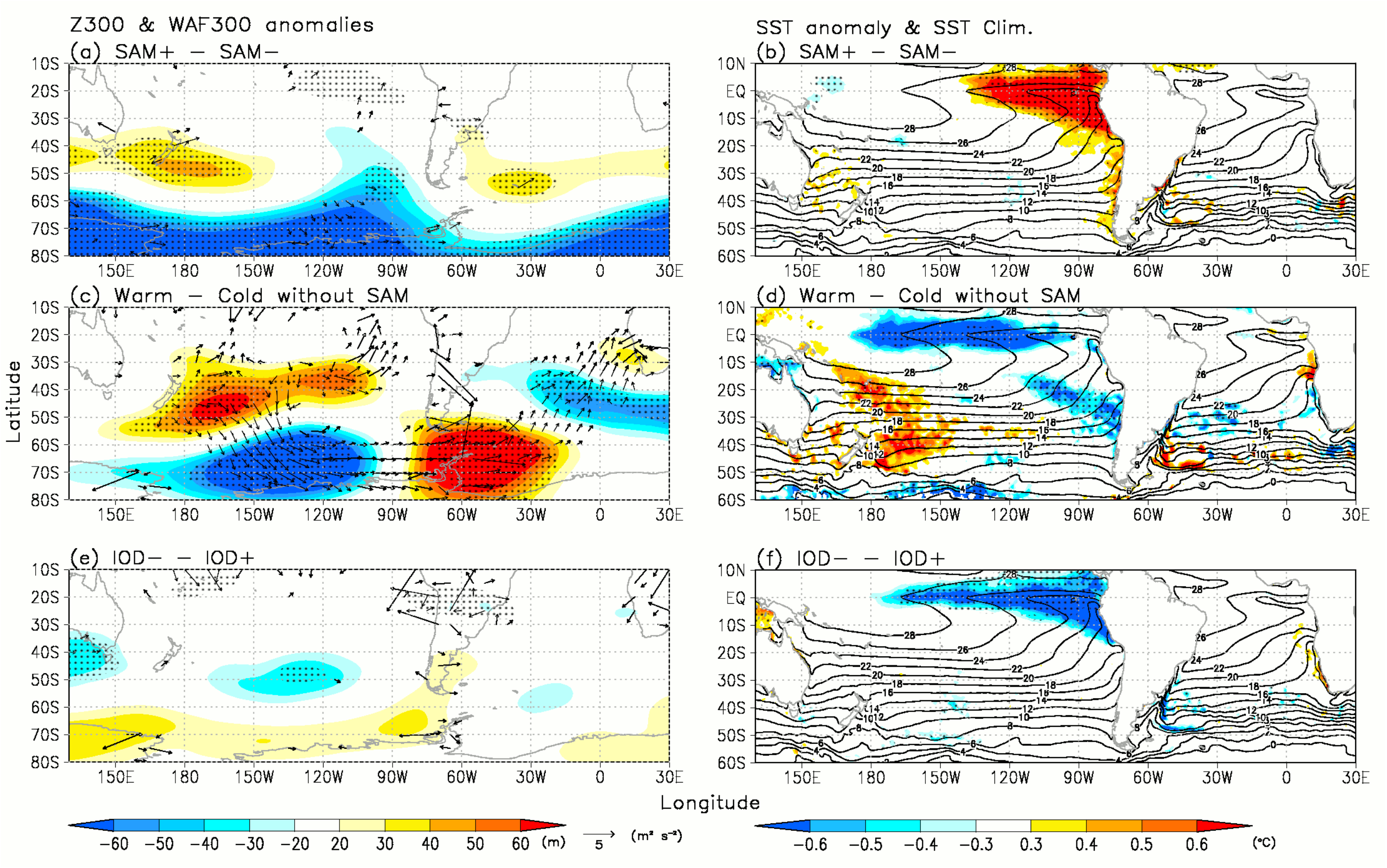

Supplementary Fig. 7. Atmospheric and ocean anomalies. Difference maps for (a) geopotential height at 300 hPa (Z300) (m: shaded) with the horizontal component of wave-activity flux anomalies (m^2/s^2: vectors) at 300 hPa (WSF300) from ref. 1 and (b) sea surface temperature (SST) differences between positive SAM and negative SAM winters. Black contours show climatological values from 1979 to 2019 for SST. Dotted areas denote significant differences exceeding the 95% confidence level. (c,d) (e,f) Same as (a,b), but for (c,d) between warm and cold winters without strong SAM and (e,f) between negative IOD and positive IOD winters.

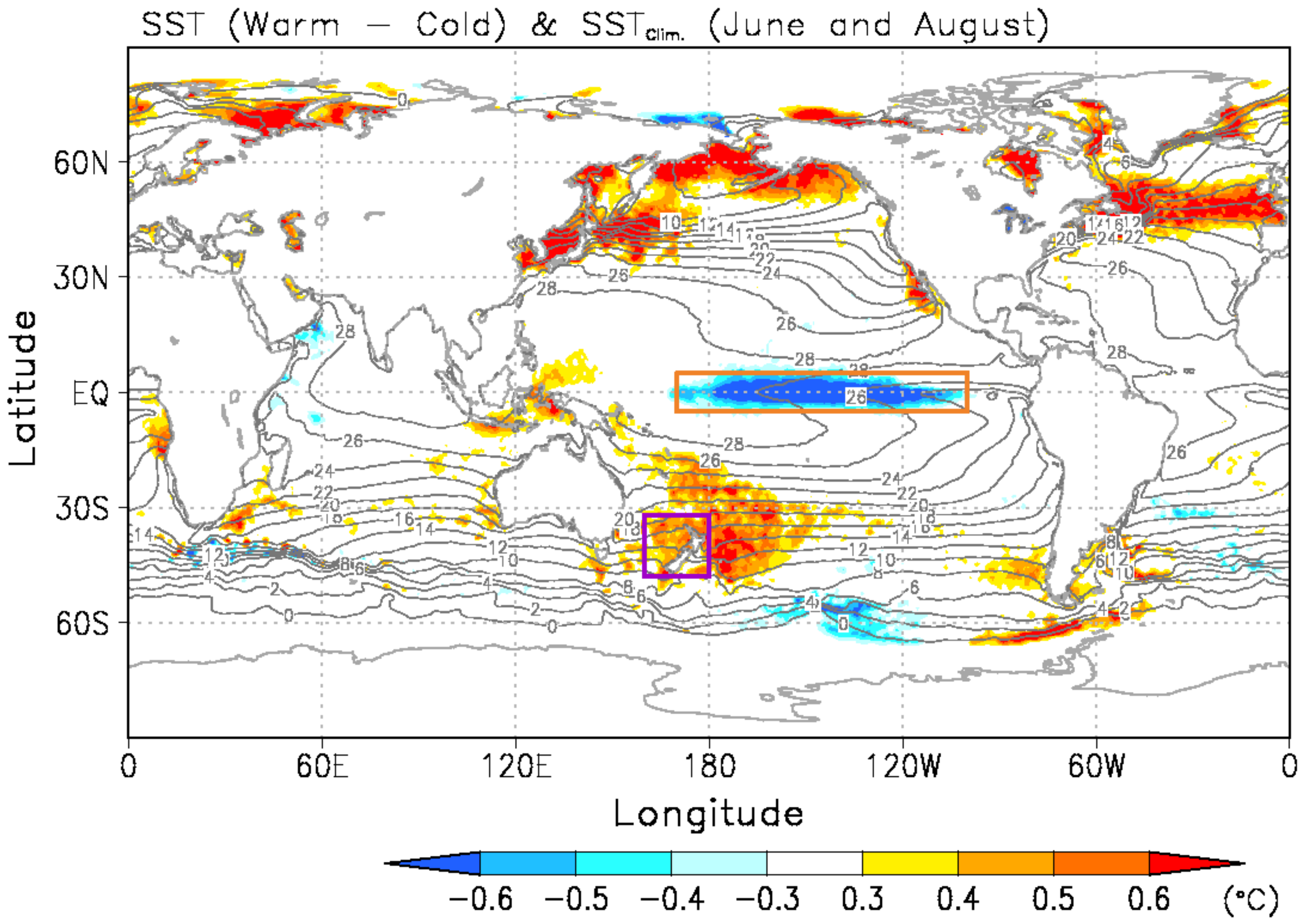

Supplementary Fig. 8. SST forcing for model experiments. Difference maps for sea surface temperature (SST) differences between warm and cold winters. Black contours show climatological values from 1979 to 2019 for SST. These results came from the ECMWF ERA5 dataset.

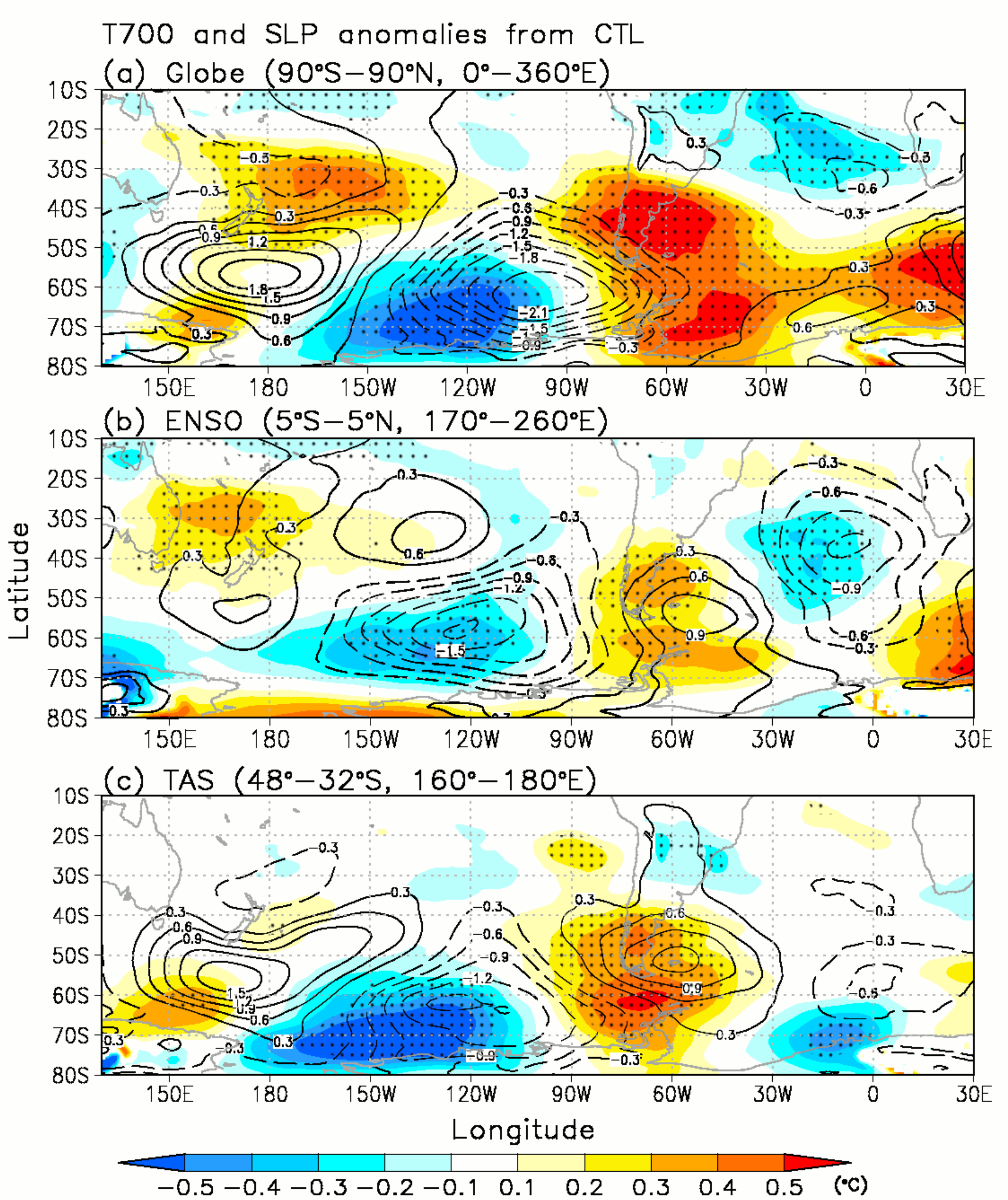

Supplementary Fig. 9. Simulated atmospheric responses to SST anomalies. Difference maps for simulated air temperature at 700 hPa (T700) (°C: shaded) and sea level pressure (SLP) (hPa: contours) for (a) between Globe and CTL, (b) between ENSO and CTL and (c) between TAS and CTL. Dotted areas denote significant differences exceeding the 95% confidence level.

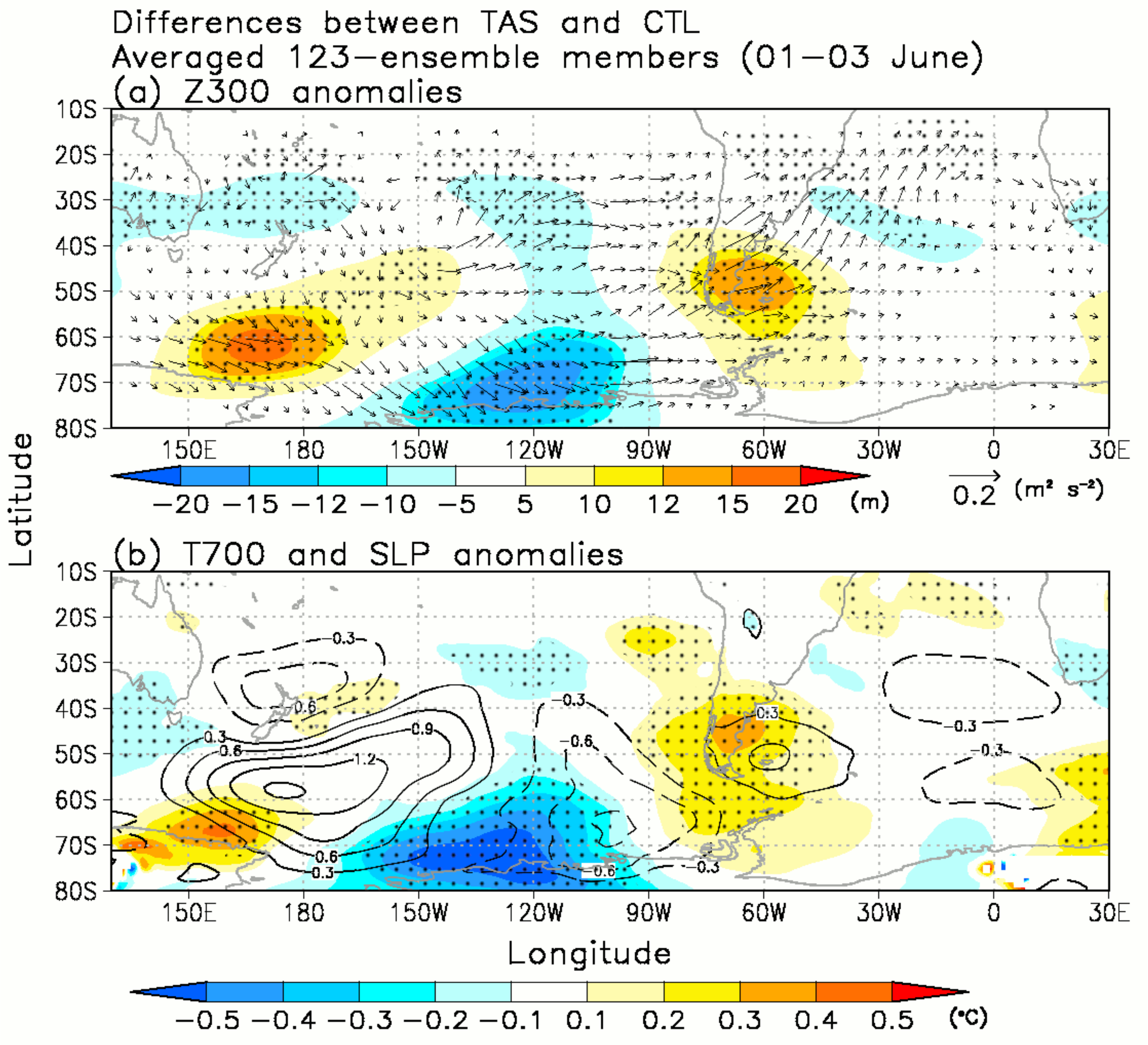

Supplementary Fig. 10. Simulated 123-ensemble members atmospheric responses to SST anomalies over the Tasman Sea. (a) Geopotential height at 300 hPa (Z300) (m: shaded) with the horizontal component of wave-activity flux anomalies (m^2/s^2: vector) at 300 hPa (WAF300) from ref. 1 and (b) air temperature at 700 hPa (T700) (°C: shaded) and sea level pressure (SLP) (hPa: contours) in TAS – CTL 123-ensemble members difference. Dotted areas denote significant differences exceeding the 95% confidence level.

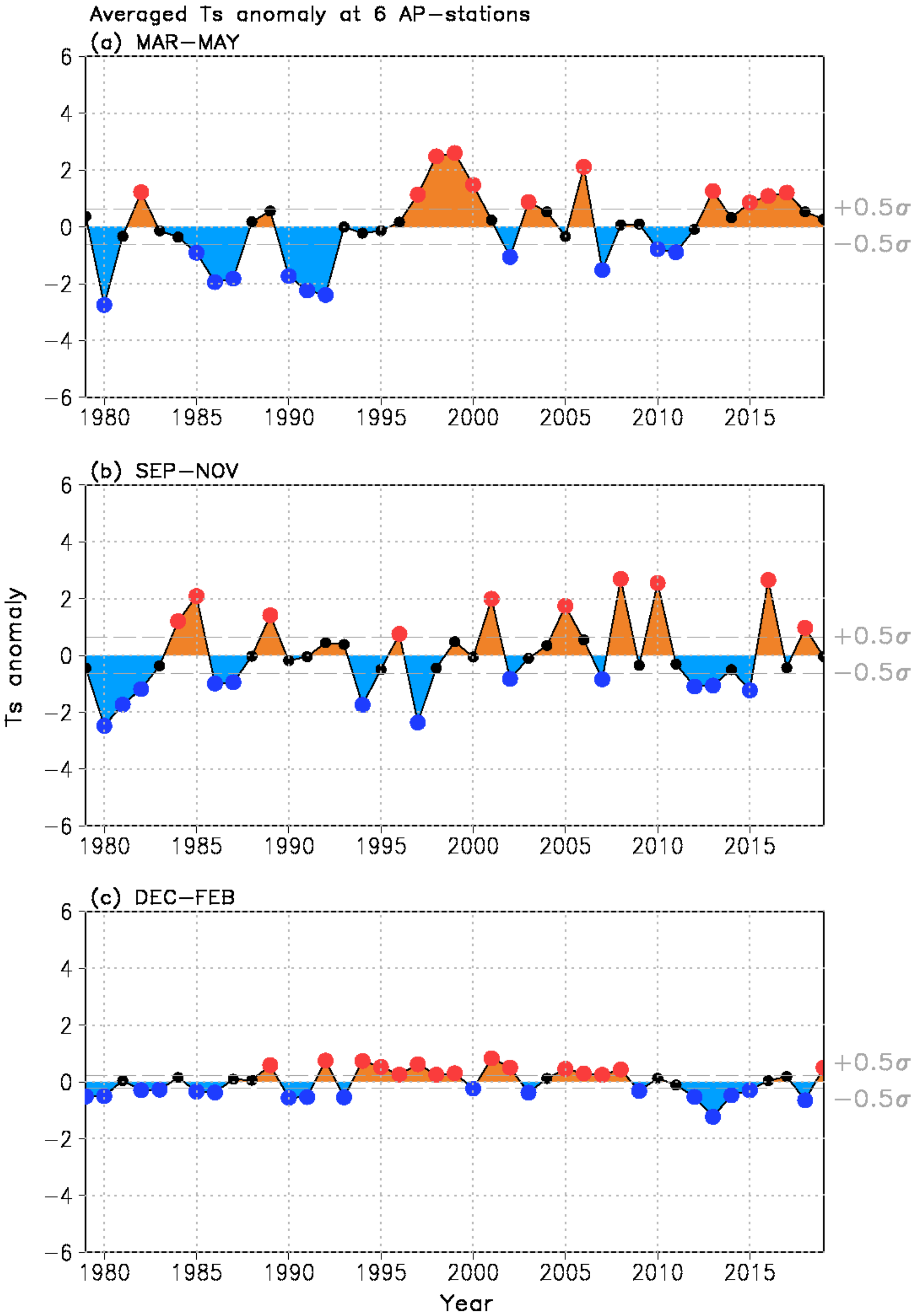

Supplementary Fig. 11. Temperature anomalies at six AP stations in all seasons. Time series of averaged surface air temperature anomalies (°C, deviation from climatology for 1979–2019) during (a) March to May, (b) September to November and (c) December to February at six Antarctic Peninsula stations (Bellingshausen, O'Higgins, Esperanza, Marambio, Vernadsky and Rothera). Red and blue dots indicate the selected warm and cold years, respectively, for each season. Dashed lines show one half standard deviation (±0.5σ).

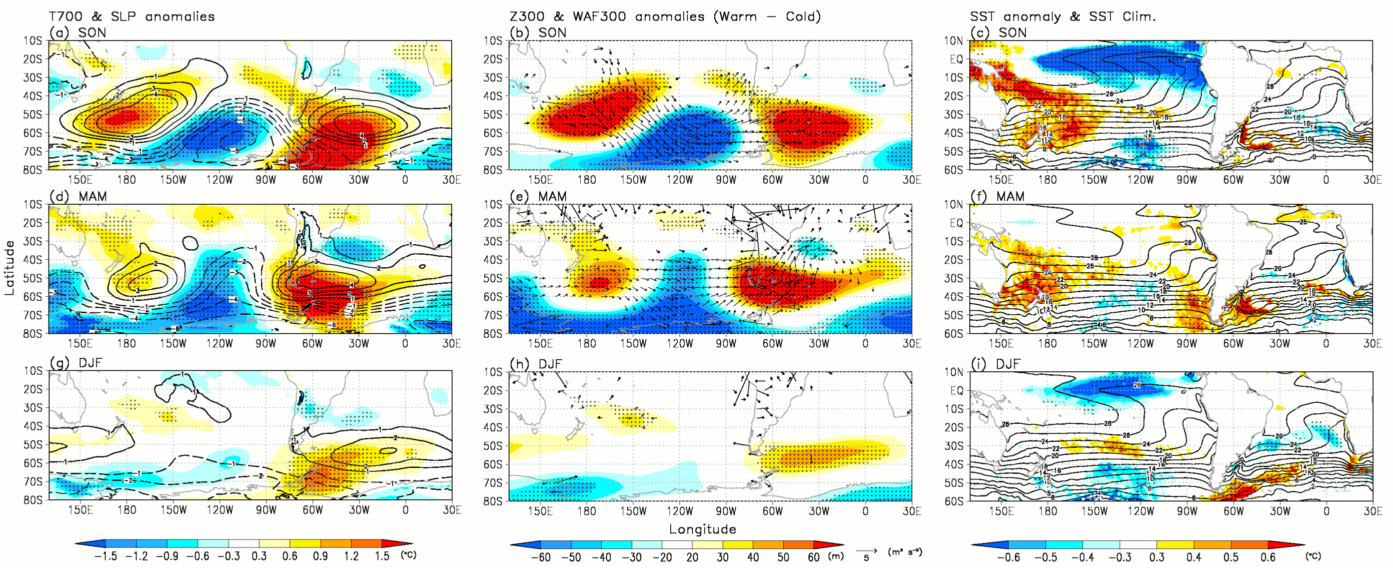

Supplementary Fig. 12. Atmospheric and ocean anomalies for spring, autumn and summer. Difference maps for (a) geopotential height at 300 hPa (Z300) (m: shaded) with the horizontal component of wave-activity flux anomalies (m^2/s^2: vector) at 300 hPa (WAF300) from ref. 1, (b) air temperature at 700 hPa (T700) (°C: shaded) and sea level pressure (SLP) (hPa: contour) and (c) sea surface temperature differences (SST) between warm and cold years for spring (September to November). Black contours show climatological values from 1979 to 2019 for SST. Dotted areas denote significant differences exceeding the 95% confidence level. (d,e,f) (g,h,i) Same as (a,b,c), but for (d,e,f), autumn (March to May) and (g,h,i) summer (December to February).

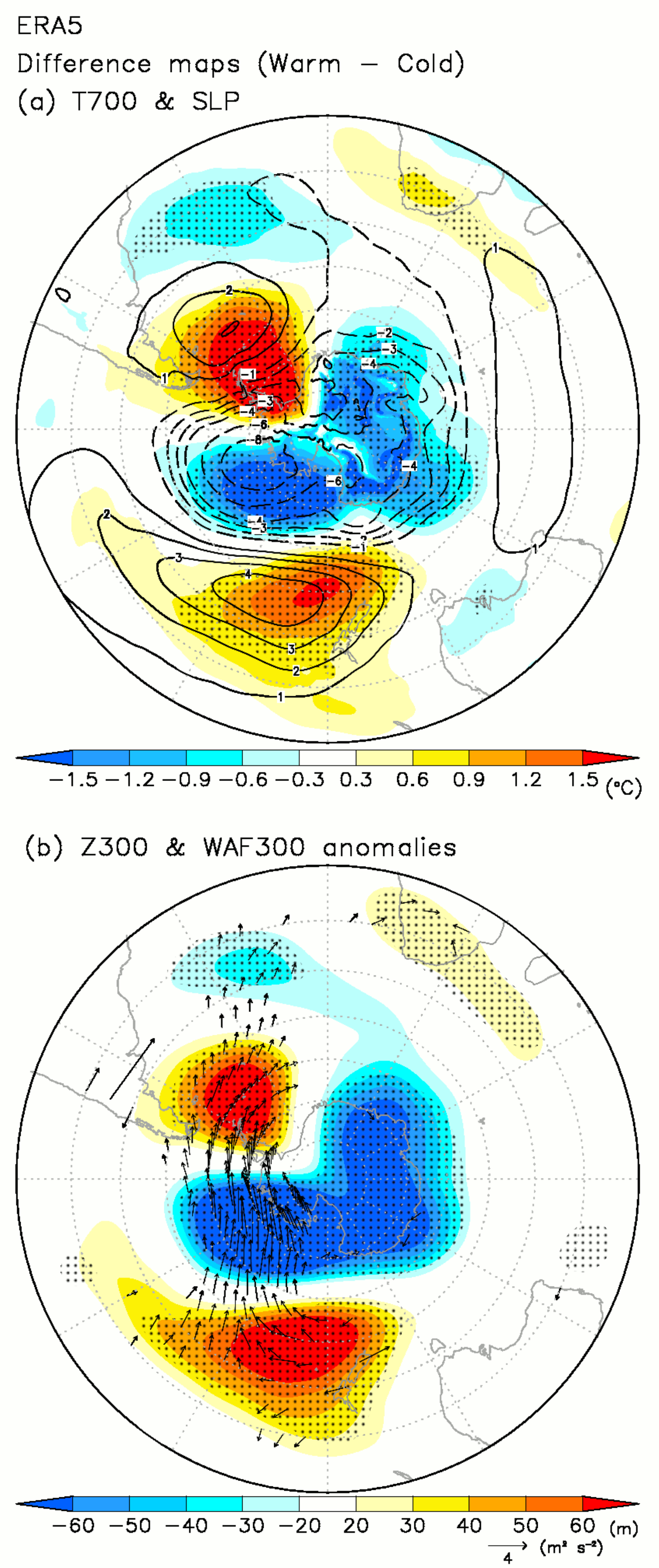

Supplementary Fig. 13. Atmospheric circulation anomalies between warm and cold winters in the ERA5 dataset. (a) Air temperature at 700 hPa (T700) (°C: shaded) and sea level pressure (SLP) (hPa: contours), and (b) geopotential height at 300 hPa (Z300) (m: shaded) differences between warm and cold winters. Arrows indicate horizontal components of wave-activity flux anomalies (m^2/s^2: vector) at 300 hPa (WAF300) from ref. 1. Dotted areas denote significant differences exceeding the 95% confidence level. These results came from the ECMWF ERA5 dataset.

Supplementary References

1. Takaya, K. & Nakamura, H. A formulation of a phase-independent wave-activity flux for stationary and migratory quasigeostrophic eddies on a zonally varying basic flow. *J. Atmos. Sci.* **58**, 608–627 (2001).
